# Structural biology and cancer

**DOI:** 10.1186/1753-6561-7-S2-K15

**Published:** 2013-04-04

**Authors:** Richard Garratt

**Affiliations:** 1 Instituto de Física de São Carlos, Universidade de São Paulo – USP, São Carlos, São Paulo, Brazil

## 

The techniques of structural biology currently play an important role in the development of novel therapeutic agents including cancer chemotherapies. X-ray crystallography has proved to be a particularly powerful tool for both the discovery of novel compounds and their subsequent development. In this review of the literature, the steps involved in the determination of a crystal structure will be described, including how to obtain good quality crystals, how to perform the X-ray diffraction experiment itself and how to solve the phase problem and refine the final structure. Emphasis will be placed on how the end-user should be aware of the limitations of the techniques involved and the potential errors present in proteins structure deposited in the Protein Data Base. Fragment-based ligand discovery and theoretical docking offer alternative approaches for focusing the structural biologist’s weaponry onto drug design problems. Of the many applications described in the literature inhibitors of HSP90 and Protein Kinase B will be used as examples of the ways in which improving specific protein-ligand interactions can be used to improve binding and pharmacokinetic properties. On the other hand, a complex structure involving a migrastatin analogue will exemplify the risks of over-interpretation of crystallographic data.

## Competing interests

There are no competing interests in this presentation.

